# Effectiveness of Influenza Vaccines in Italy in the 2024/2025 Season: A Nationwide, Test‐Negative Design Study Based on Surveillance Records

**DOI:** 10.1111/irv.70252

**Published:** 2026-04-30

**Authors:** Daniel Cifo, Alberto Mateo‐Urdiales, Simona Puzelli, Marzia Facchini, Sara Piacentini, Emanuela Giombini, Benedetta Bellini, Massimo Fabiani, Patrizio Pezzotti, Paola Stefanelli, Anna Teresa Palamara, Antonino Bella, the RespiVirNet network

**Affiliations:** ^1^ Escuela Nacional de Sanidad Instituto de Salud Carlos III Madrid Spain; ^2^ Programa de Doctorado en Ciencias Biomédicas y Salud Pública Instituto Mixto de Investigación UNED‐ENS Madrid Spain; ^3^ Department of Infectious Diseases Istituto Superiore di Sanità Rome Italy

**Keywords:** epidemiology, influenza, surveillance, vaccine effectiveness

## Abstract

The 2024/2025 influenza season in Italy was characterised by cocirculation of influenza A and B viruses. Monitoring influenza vaccine effectiveness (IVE) remains essential to guide public health policy due to antigenic shifts and varying strain circulation. In this study, we aimed to estimate IVE in primary care and hospital settings during the 2024/2025 season in Italy. A nationwide test‐negative control design was used. Data were collected from the RespiVirNet surveillance system, encompassing both primary care and hospital settings. A total of 8842 participants were included in the primary care analysis and 2492 in the hospital analysis. Adjusted vaccine effectiveness (aVE) against any influenza virus was 69.0% (95% CI: 60.4%–75.9%) in primary care and 62.3% (95% CI: 45.9%–74.2%) in hospital settings. Effectiveness was highest among individuals under 18 years and decreased with age. Influenza B showed the highest aVE (83.6% in primary care; 95.0% in hospital settings), particularly among younger individuals. aVE for influenza A subtypes was lower, with A H1N1pdm09 (54.0% in primary care; 63.8% in hospital) and A H3N2 (60.1% in primary care; 45.5% in hospital) showing moderate effectiveness. The results suggest that influenza vaccines were effective in preventing medically attended influenza disease, both in primary care and hospital settings. These findings provide valuable insights for public health planning and vaccine policy in Italy.

## 1 Introduction

Seasonal influenza remains a significant public health threat, causing substantial morbidity and mortality [1]. Its epidemiology is based on the ability of influenza viruses to change the antigenic specificity of the surface glycoproteins, the haemagglutinin (H) and the neuraminidase (N), which may lead either to minor (antigenic drift) or, sometimes, major (antigenic shift) modifications, respectively responsible for epidemics and pandemics. These changes enable the virus to evade human humoral immunity, making individuals susceptible to infection despite prior exposure in previous seasons [2]. With few exceptions, notably during the COVID‐19 pandemic [3], influenza epidemic seasons typically coincide with the cold months in both the northern and southern hemispheres.

Seasonal flu vaccination is the best public health intervention to prevent influenza‐related sequelae, including death, and it is considered cost‐effective, despite suboptimal coverage [4]. Like natural immunity, antigenic variability affects the effectiveness of influenza vaccines (IVE) across different sessions. Consequently, the annual development of new vaccines is necessary to target the most likely circulating strains [5]. As vaccine composition is determined before the start of the season, it is essential to evaluate IVE each year to monitor their performance in real‐world scenarios [6]. During the 2024/2025 season in the northern hemisphere, the World Health Organization (WHO) recommended, for egg‐based vaccines, the following influenza virus strains: an A/Victoria/4897/2022 (H1N1)pdm09‐like, an A/Thailand/8/2022 (H3N2)‐like and a B/Austria/1359417/2021 (B/Victoria lineage)‐like virus for trivalent vaccines; for cell culture or recombinant‐based vaccines, an A/Wisconsin/67/2022 (H1N1)pdm09‐like and an A/Massachusetts/18/2022 (H3N2)‐like virus, with the same influenza B virus component as for egg‐based vaccines. For both egg‐ and cell culture–, recombinant protein– or nucleic acid–based vaccines, the WHO recommended inclusion of an additional B/Phuket/3073/2013 (B/Yamagata lineage)‐like virus for quadrivalent vaccines [7].

Interim vaccine effectiveness results from studies in the European Union (EU) and in the United States during the 2024/2025 season suggest a moderate effectiveness of influenza vaccine, higher for influenza B than for A H1N1pdm09 or A H3N2, and higher in the younger age groups [8, 9].

The EU studies were carried out in a context where the proportion of influenza A cases (63%) was similar to that observed in Italy at the end of the season (67%) [10], whereas in the United States, the proportion of influenza A cases was significantly higher (97%). Among influenza A cases, in Italy, we observed a higher percentage of H3N2 cases (46% of all influenza A cases) compared with EU countries participating in the studies (20%) and slightly lower than the proportion observed in the United States (52%). Either in the EU, in the United States and in Italy, the majority of A H1N1pdm09 viruses belonged to the 5a.2a clade, and the majority of influenza A H3N2 viruses belonged to the 2a.3a.1 clade. Equally, characterised influenza B viruses in the EU countries and in Italy belonged to clade V1A.3a.2 [11].

The objective of this study was to estimate IVE in Italy for the complete 2024/2025 epidemic season, in primary care and hospital settings, providing evidence that will serve as guidance for public health authorities.

## 2 Methods

### 2.1 Data Source

Data for this study were retrieved from the Italian respiratory surveillance system (RespiVirNet) [12]. For the primary care analysis, we used the sentinel surveillance system, which is based on the voluntary participation of 404 general practitioners and paediatricians. During the season, clinicians are asked to test a sample of the patients that they see meeting the influenza‐like illness (ILI) case definition. There is no indication on how the sample should be selected, leaving that decision at the clinician's judgment. Despite being a national surveillance system, there is in an unequal distribution of participating clinicians across the Italian regions (see Table S1).

For the hospital analysis, we used data from the Italian laboratory network (RespiVirNet). This network of 27 regional laboratories collects data on all tests for influenza and other respiratory viruses and sends the information to the laboratory of the WHO National Influenza Centre at the ISS (NIC‐ISS) [13]. Table S2 shows the number of tests processed in each Italian region during the season.

Data collected included region, age, sex, symptom onset date, comorbidities, vaccination status (date and type), antiviral therapy, epidemiological week and laboratory diagnostic test results (date, method and outcome). The diagnostic methods were based on real‐time polymerase chain reaction (RT‐PCR) assays. Further strain characterisation was carried out by the RespiVirNet laboratory network and by the NIC‐ISS, for influenza A H1N1pdm09, A H3N2 and influenza B/Victoria lineage. The B/Yamagata lineage has not been detected since March 2020.

### 2.2 Study Design, Enrolment Criteria

A test‐negative study was conducted to estimate IVE in Italy during the 2024–2025 epidemic season. In the primary care setting, participants are recruited among patients seeking healthcare for ILI. The inclusion criteria for the study were to seek consultation from a practitioner with Influenza‐like illness (ILI) symptoms from Week 46 of 2024 to Week 17 of 2025, having a nasal or throat mucus sample within 7 days after symptom onset and a registered vaccination status. The Italian respiratory surveillance protocol uses the ILI case‐definition proposed by the European Centre for Disease Prevention and Control (ECDC) in concordance with the Decision of the EU Commission 2018/945 [14].

In the hospital setting, the network of laboratories sends test results and key variable information on those being tested for influenza in‐hospital.

In both settings, participants are classified as cases or controls based on the result of a laboratory‐confirmed influenza diagnostic test. IVE is then estimated by calculating one minus the odds ratio of vaccination (OR_vac_) between the two groups [15].

Participants were excluded if they had received vaccination less than 15 days before symptom onset, were vaccinated before the start of the seasonal vaccination campaign, were tested more than 7 days after symptom onset, or were under 6 months of age (ineligible for vaccination).

### 2.3 Statistical Analysis

Basic epidemiologic measures, including case and control counts and rates, were calculated per influenza strain and epidemic week. A descriptive stratified analysis was performed according to case and control status and, for cases, by influenza strain.

**FIGURE 1:**
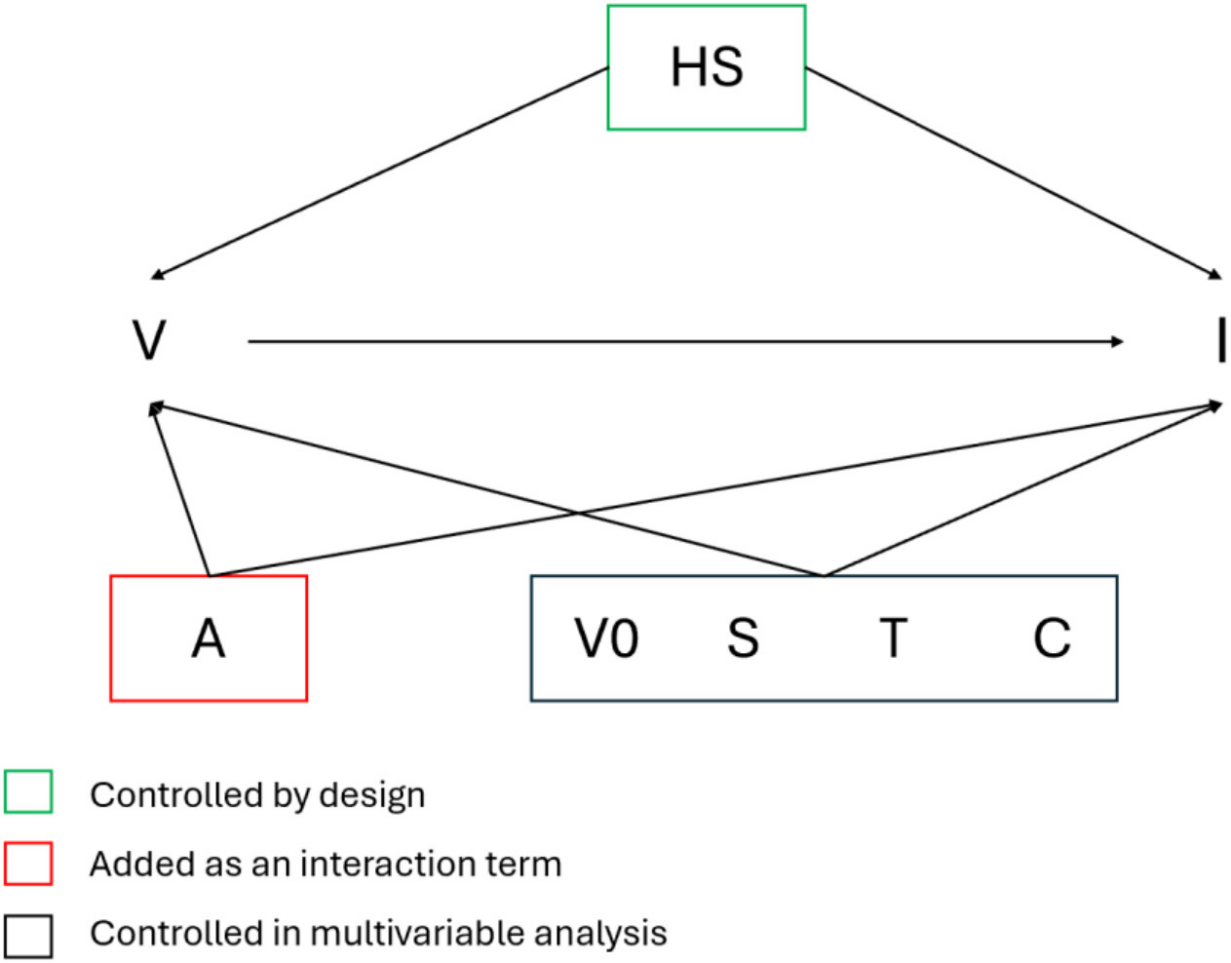
DAG of the causal framework of the study. HS = healthcare seeking behaviour, V = flu vaccination status, I = influenza detected, A = age, V0 = previous flu vaccination status, S = sex, T = calendar time, C = comorbidities.

For the multivariable analysis, we drew a directed acyclic graph (DAG) illustrating the causal framework of the study to ascertain the possible confounders and effect modifiers in the association between vaccination and influenza infection in primary care and hospital settings (Figure 1). Logistic regression models were used to obtain adjusted OR_vac_ in cases (both overall and by influenza strain) compared to controls, including potential confounders such as sex, age group, calendar time (week), vaccination in the previous season, and comorbidities as covariates. OR_vac_ by age group (i.e., < 18, 18–64 and 65+ years) were obtained including into the models an interaction term between age group and influenza test result. IVE was estimated by calculating 1 minus the OR_vac_. All statistical analyses were performed using the R statistical software, version 4.5.0 [16].

## 3 Results

**FIGURE 2:**
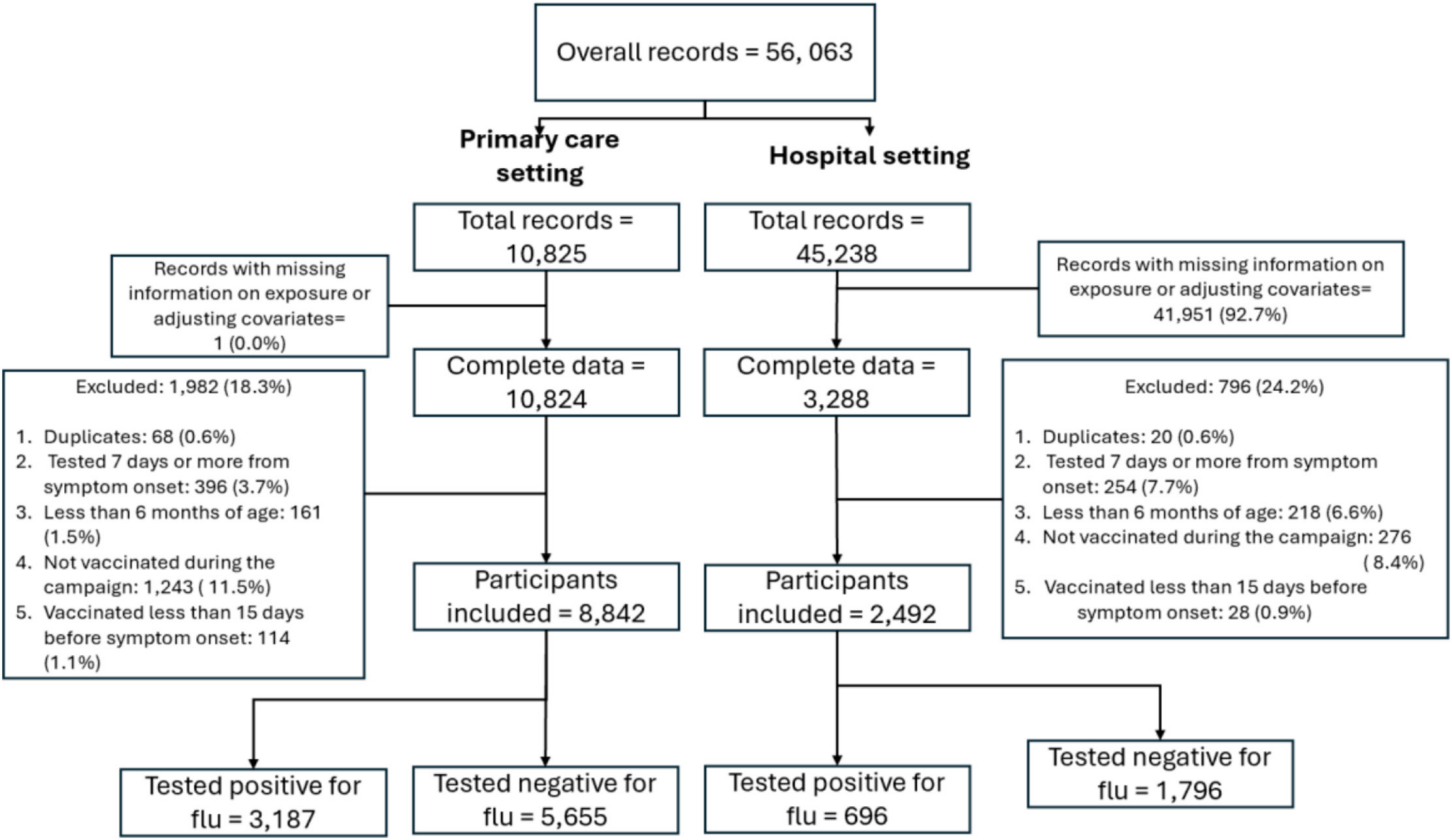
Flowchart of participants of the study.

**FIGURE 3:**
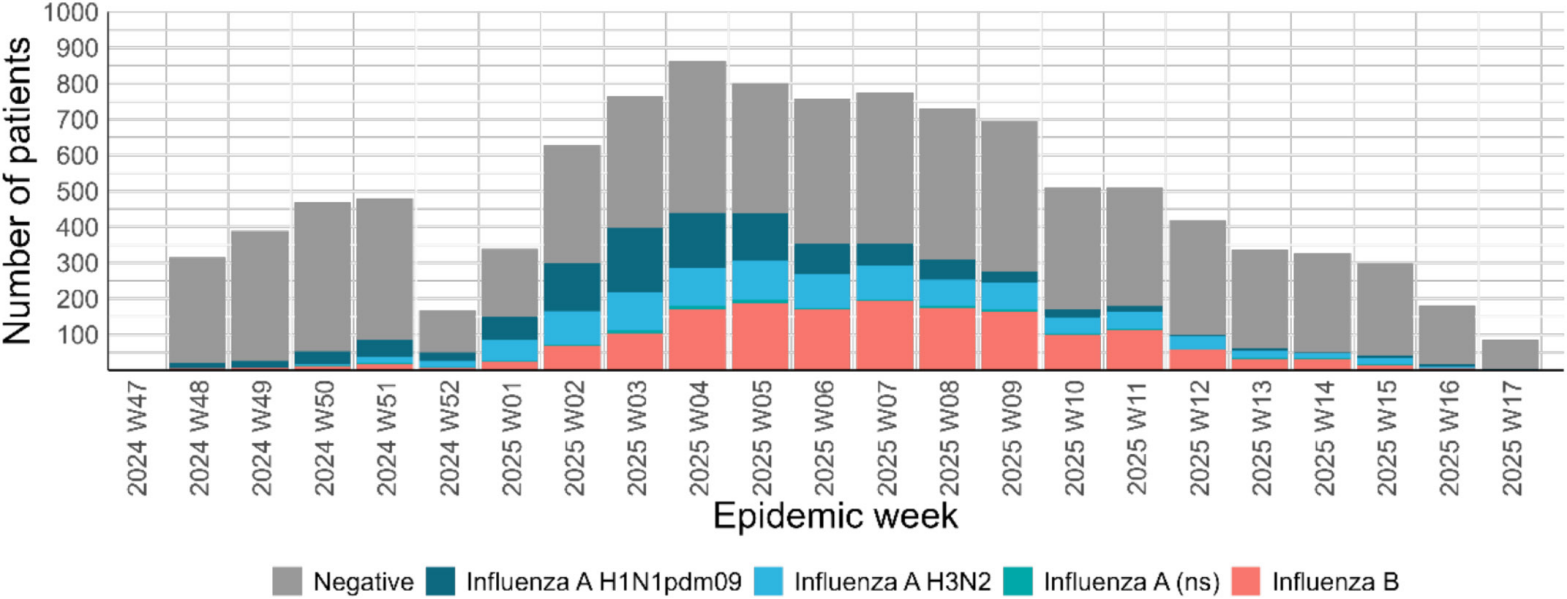
Epidemic curve with the number of influenza‐like illness among the study population by week of onset, influenza test and subtype. Italy, 2024–2025.

After exclusions, we included 8842 participants in the analysis of IVE in primary care settings and 2492 in the analysis of IVE in hospital settings (Figure 2). Among included participants in the primary care setting, 3187 (36%) tested positive for influenza. Of these, 53% tested positive for influenza A (28% for H1N1pdm09, 23% for H3N2, and 2% were not characterised) and 47% tested positive for influenza B. In the hospital setting, 696 (28%) tested positive for influenza. Of these, 76% tested positive for influenza A (31% for H1N1pdm09, 42% for H3N2, and 3% were not typified) (Table 1). Among study participants, the ILI peak occurred in Week 4 of 2025. The highest influenza positivity rate was observed in Week 4 of 2025 at 40.2% (Figure 3).

**TABLE 1:** Characteristics of the participants included in the study.

	Influenza test negative (*n* = 5655) *n* (%)	Influenza test positive (*n* = 3187)			
		Influenza A H1N1pdm09 (*n* = 889) *n* (%)	Influenza A H3N2 (*n* = 743) *n* (%)	Influenza A no subtype (*n* = 58) *n* (%)	Influenza B (*n* = 1497) *n* (%)
Age; median (IQR)	21 (5, 45)	19 (5, 49)	19 (5, 39)	38 (4, 56)	12 (7, 32)
Age group (years)					
< 18	2697 (48%)	440 (49%)	362 (49%)	23 (40%)	946 (63%)
18–64	2634 (47%)	413 (46%)	348 (47%)	30 (52%)	540 (36%)
65+	324 (5.7%)	36 (4.0%)	33 (4.4%)	5 (8.6%)	11 (0.7%)
Sex					
Female	2958 (52%)	454 (51%)	364 (49%)	27 (47%)	734 (49%)
Male	2697 (48%)	435 (49%)	379 (51%)	31 (53%)	763 (51%)
Chronic conditions					
No	4934 (87%)	795 (89%)	662 (89%)	47 (81%)	1396 (93%)
Yes	721 (13%)	94 (11%)	81 (11%)	11 (19%)	101 (6.7%)
Interval onset to swab (days)					
0–1	1568 (28%)	235 (26%)	213 (29%)	13 (22%)	289 (19%)
2–4	3444 (61%)	568 (64%)	475 (64%)	37 (64%)	1010 (67%)
5–7	643 (11%)	86 (9.7%)	55 (7.4%)	8 (14%)	198 (13%)
Vaccination status					
2024–2025 season					
No	5087 (90%)	838 (94%)	702 (94%)	54 (93%)	1472 (98%)
Yes	568 (10%)	51 (5.7%)	41 (5.5%)	4 (6.9%)	25 (1.7%)
2023–2024 season					
No	4788 (85%)	785 (88%)	658 (89%)	47 (81%)	1387 (93%)
Yes	507 (9.0%)	60 (6.7%)	45 (6.1%)	5 (8.6%)	29 (1.9%)
Unknown	360 (6.4%)	44 (4.9%)	40 (5.4%)	6 (10%)	81 (5.4%)
2022–2023 season					
No	4816 (85%)	789 (89%)	655 (88%)	50 (86%)	1371 (92%)
Yes	422 (7.5%)	52 (5.8%)	44 (5.9%)	2 (3.4%)	35 (2.3%)
Unknown	417 (7.4%)	48 (5.4%)	44 (5.9%)	6 (10%)	91 (6.1%)
Target group for vaccination					
No	3358 (59%)	549 (62%)	476 (64%)	33 (57%)	1099 (73%)
Yes	2296 (41%)	340 (38%)	267 (36%)	25 (43%)	398 (27%)
Unknown	1 (< 0.1%)	0 (0%)	0 (0%)	0 (0%)	0 (0%)

Hospital setting					
	Influenza test negative (*n* = 1796) *n* (%)	Influenza test positive (*n* = 696)			
		Influenza A H1N1pdm09 (*n* = 218) *n* (%)	Influenza A H3N2 (*n* = 292) *n* (%)	Influenza A no subtype (*n* = 20) *n* (%)	Influenza B (*n* = 166) *n* (%)
Age; median (IQR)	15 (2, 61)	59 (7, 77)	38 (4, 69)	47 (30, 54)	7 (3, 11)
Age group (years)					
< 18	916 (51%)	72 (33%)	133 (46%)	3 (15%)	139 (84%)
18–64	482 (27%)	44 (20%)	68 (23%)	13 (65%)	20 (12%)
65+	398 (22%)	102 (47%)	91 (31%)	4 (20%)	7 (4.2%)
Sex					
Female	856 (48%)	113 (52%)	133 (46%)	10 (50%)	81 (49%)
Male	940 (52%)	105 (48%)	159 (54%)	10 (50%)	85 (51%)
Chronic conditions					
No	902 (50%)	76 (35%)	132 (45%)	4 (20%)	114 (69%)
Yes	712 (40%)	117 (54%)	146 (50%)	10 (50%)	37 (22%)
Unknown	182 (10%)	25 (11%)	14 (4.8%)	6 (30%)	15 (9.0%)
Interval onset to swab (days)					
0–1	699 (39%)	88 (40%)	93 (32%)	10 (50%)	43 (26%)
2–4	921 (51%)	88 (40%)	151 (52%)	7 (35%)	76 (46%)
5–7	176 (9.8%)	42 (19%)	48 (16%)	3 (15%)	47 (28%)
Vaccination status					
2024–2025 season					
No	1568 (87%)	201 (92%)	265 (91%)	19 (95%)	165 (99%)
Yes	228 (13%)	17 (7.8%)	27 (9.2%)	1 (5.0%)	1 (0.6%)
2023–2024 season					
No	890 (50%)	102 (47%)	180 (62%)	12 (60%)	119 (72%)
Yes	157 (8.7%)	28 (13%)	28 (9.6%)	0 (0%)	2 (1.2%)
Unknown	749 (42%)	88 (40%)	84 (29%)	8 (40%)	45 (27%)
2022–2023 season					
No	845 (47%)	91 (42%)	169 (58%)	12 (60%)	108 (65%)
Yes	132 (7.3%)	21 (9.6%)	26 (8.9%)	0 (0%)	2 (1.2%)
Unknown	819 (46%)	106 (49%)	97 (33%)	8 (40%)	56 (34%)
Target group for vaccination					
No	725 (40%)	57 (26%)	83 (28%)	14 (70%)	64 (39%)
Yes	1043 (58%)	147 (67%)	206 (71%)	5 (25%)	92 (55%)
Unknown	28 (1.6%)	14 (6.4%)	3 (1.0%)	1 (5.0%)	10 (6.0%)

Abbreviation: IQR = interquartile range.

Among participants attending primary care, those positive for influenza B were more likely to be < 18 years of age and to not have chronic conditions compared with those testing positive for influenza A or testing negative. There were no substantial differences by sex and interval onset to swab. Cases were less likely to be vaccinated both in the 2024/2025 season and in the previous 2 years.

In hospital settings, influenza B cases were the youngest compared with influenza A cases and those testing negative. Influenza A cases were older than those testing negative and more likely to have chronic conditions. Also in this setting, cases were less likely to be vaccinated.

### 3.1 Vaccine Effectiveness

**FIGURE 4:**
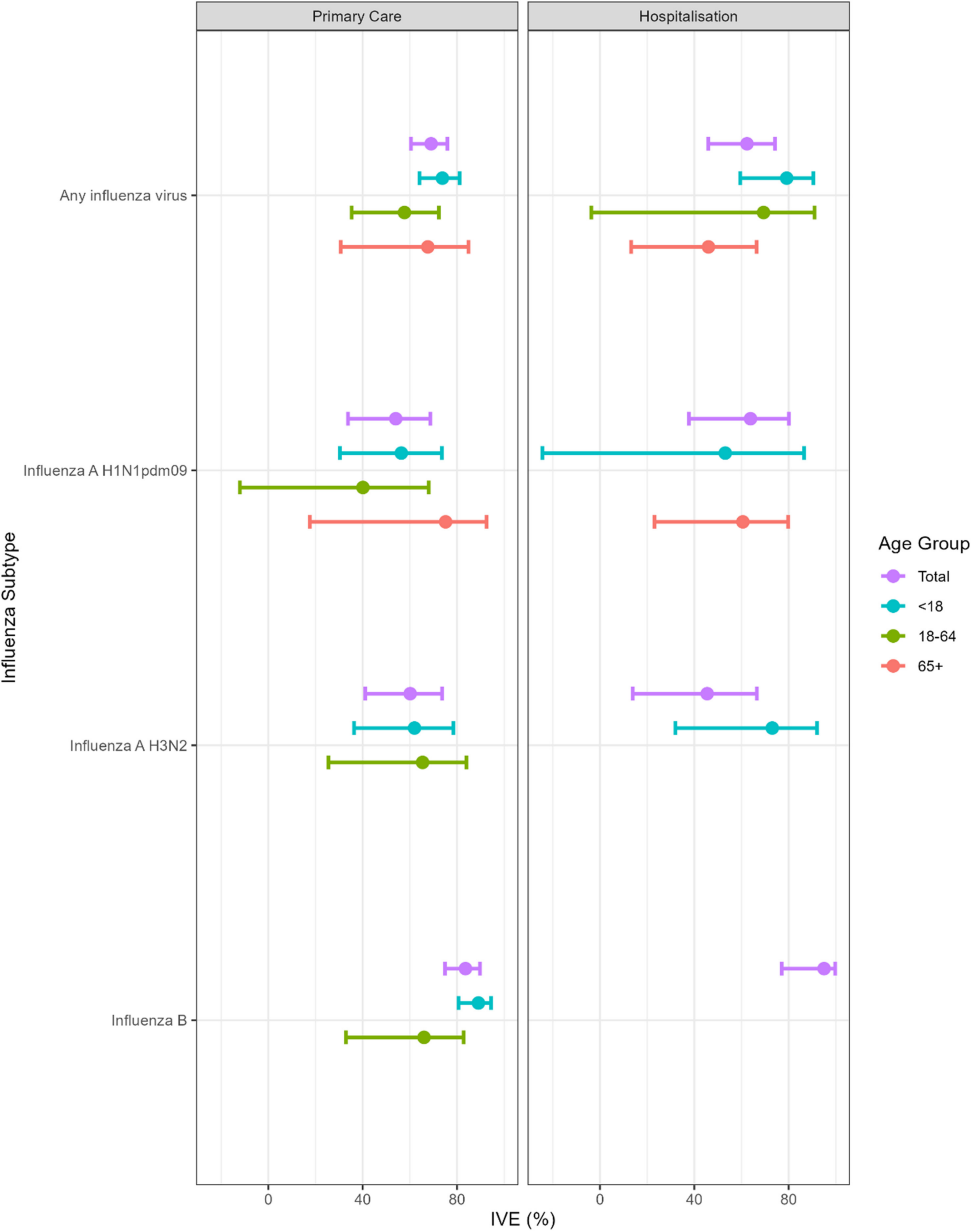
Adjusted vaccine effectiveness against (adjusted for age, sex, previous vaccination status, calendar time and presence of comorbidities) any influenza virus, and by virus type and subtype. Overall and stratified by age group in Italy, during the 2024/2025 season.

In the primary care setting, overall adjusted vaccine effectiveness (aVE) against any influenza virus was 69.0% (95% confidence interval [CI]: 60.4%–75.9%) (Table 2 and Figure 4). Effectiveness was higher in the < 18 age group (aVE: 73.8%; 95% CI: 64.0%–81.1%) and lowest in the 18–64 age group (57.7%; 95% CI: 35.3%–72.3%), though the difference was not statistically significant. Among influenza subtypes, overall aVE was significantly higher for influenza B (83.6%; 95% CI: 74.9%–89.7%) than for A H1N1pdm09 (54.0%; 95% CI: 33.8%–68.6%) (*p* < 0.001) and for A H3N2 (60.1%; 95% CI: 41.1%–73.7%) (*p* = 0.002). According to age group and influenza subtype, the highest aVE was observed in the < 18 age group for influenza B (significantly higher than in the 18–64 age group), in the 18–64 age group for influenza A H3N2 and in those aged 65+ for influenza A H1N1pdm09.

**TABLE 2:** Crude and adjusted vaccine effectiveness against any influenza virus and by virus type and subtype. Overall and stratified by age group in Italy, during the 2024/2025 season.

Primary care	Age group (years)	IVE (crude)	95% CI	IVE (adj.)^a^	95% CI
Any influenza virus	Total	64.7	56.9–71.2	69.0	60.4–75.9
	< 18	67.5	57.4–75.5	73.8	64.0–81.1
	18–64	54.3	34.6–68.9	57.7	35.3–72.3
	65+	56.4	25.7–75.3	67.6	30.7–84.8
Influenza A H1N1pdm09	Total	45.5	27.5–59.9	54.0	33.8–68.6
	< 18	37.6	9.3–58.6	56.4	30.4–73.5
	18–64	38.0	−3 to 65.4	40.1	−12.1 to 68.0
	65+	71.6	34.5–89.6	75.1	17.6–92.5
Influenza A H3N2	Total	47.7	28.4–62.8	60.1	41.1–73.7
	< 18	50.1	22.4–69.7	61.9	36.3–78.4
	18–64	56.3	18.5–79.5	65.4	25.5–83.9
	65+	19.0	−67.9 to 62.5	36.3	−84.5 to 78.0
Influenza B	Total	84.8	77.7–90.1	83.6	74.9–89.7
	< 18	89.0	81.2–94.2	89.1	80.6–94.4
	18–64	65.8	39.4–82.7	66.0	32.8–82.8
	65+	68.5	−24.7 to 95.2	82.4	−46.4 to 97.9

Hospital setting	Age group (years)	IVE (crude)	95% CI	IVE (adj.)^a^	95% CI
Any influenza virus	Total	51.3	33–65.4	62.3	45.9–74.2
	< 18	77.0	56.3–89.3	79.1	59.4–90.5
	18–64	71.3	18.3–93.2	69.3	−3.7 to 90.9
	65+	40.4	9–61.7	46.0	13.2–66.4
Influenza A H1N1pdm09	Total	41.8	5.5–66.4	63.8	37.7–80.1
	< 18	49.2	−26.3 to 84.8	53.1	−24.4 to 86.5
	18–64	—	—	—	—
	65+	56.5	21.3–77.6	60.6	23.1–79.8
Influenza A H3N2	Total	29.9	−4.8 to 54.9	45.5	13.9–66.5
	< 18	73.2	34.6–91.9	73.0	32–92
	18–64	37.2	−81.6 to 85.2	34.3	−128.9 to 81.2
	65+	16.1	−42.5 to 52.4	28.0	−30.7 to 60.3
Influenza B	Total	95.8	81.3–99.8	95.0	77–99.7
	< 18	100.0	90–100	—	—
	18–64	—	—	—	—
	65+	50.3	−195.6 to 97.4	−9.2	−876.9 to 87.8

^a^ Adjusted for age, sex, previous vaccination status, calendar time and presence of comorbidities.

In the hospital setting, overall aVE against any influenza virus was 62.3% (95% CI: 45.9%–74.2%) (Table 2 and Figure 4). A decreased trend in aVE with age was observed in the hospital setting, with the highest aVE against any influenza virus in the < 18 age group (79.1%; 95% CI: 59.4%–90.5%) and the lowest in the 65+ (46.0%; 95% CI: 13.2%–66.4%), though differences were not statistically significant. In this setting, aVE was higher for influenza B (95%; 95% CI: 77%–99.7%) than for influenza A H1N1pdm09 (aVE: 63.8%; 95% CI: 37.7%–80.1%) (*p* = 0.059) and for influenza A H3N2 (aVE: 45.5%; 95% CI: 13.9%–66.5%) (*p* = 0.021).

## 4 Discussion

### 4.1 Summary of Findings

In Italy, during the 2024/2025 season people testing positive for influenza in the primary care setting were 69% less likely to be vaccinated than those testing negative. Vaccine effectiveness was slightly lower (62.3%) in the hospital setting. This season, vaccine effectiveness was higher for influenza B, which affected predominantly those under 18 years of age than for influenza A H1N1pdm09 or influenza A H3N2. We did not observe relevant differences in vaccine effectiveness against influenza A H1N1pdm09 and influenza A H3N2.

### 4.2 Comparison With Other Studies and Possible Explanations

We found generally higher IVE estimates against any influenza than those reported in the interim results from the United States or Canada [9, 17]. This could be at least partially explained by the low levels of influenza B circulation in North America with respect to Italy, as IVE was highest against this type of influenza. In fact, our IVE estimates against A H1N1pdm09 and A H3N2 are in line with the interim results from Canada [17]. Our overall estimates are also generally higher than the interim reports in the European Union, where we observed a similar cocirculation of influenza A and influenza B virus [8]. We are unsure of the reasons behind these differences in IVE, though it will be clearer once the final estimates for the season in the EU are available. We did, however, find similar patterns of IVE estimates by age and subtype. Similarly to the 2024/2025 interim results from the EU, Denmark, and the United Kingdom, we found a higher IVE for influenza B than for influenza A [8, 9]. A higher effectiveness for influenza B was also observed during the 2022/2023 season in the EU [18]; and in the United States in the 2023/2024 season [19]. This season, the circulating influenza B strains observed in Italy belonged to B/Victoria‐lineage, clade V.1A.3a.2, characterised by HA substitutions A127T, P144L and K203R; within V1A.3a.2 clade, viruses fell in the C.5 subclade defined by the additional D197E genetic change. These matched the vaccine composition that could, at least, partially explain the effectiveness observed. However, there are possible factors that could also explain the higher effectiveness observed for influenza B. For example, this season in Italy, influenza B affected almost exclusively people under 65 years of age. It is possible that younger individuals, who are less likely to be affected by chronic conditions, have a stronger immune response to vaccination [20]. This might also explain the decreasing trend in influenza effectiveness with increasing age observed in the hospital setting.

With regards to A H1N1pdm09 viruses, the majority belonged to the 5a.2a clade, like during the 2023/2024 season and the vaccine composition remained unchanged. This season we found that the majority of 5a.2a viruses belonged to the subclade C.1.9, characterised by the K169Q HA substitution. The vaccine seemed to keep its moderate effectiveness against A H1N1pdm09, as the estimate we found this season in the primary care setting (54%) is similar to those reported the previous season in studies carried out in Italy and the EU (in both cases around 50%) [21, 22, 23].

The A H3N2 viruses circulating in the 2024/2025 season belonged to the 2a.3a.1 clade, and particularly to the J.2 subclade, characterised by the N122D and K276E HA mutations. As reported by WHO, post‐infection ferret antisera raised against H3N2 vaccine viruses showed reduced reactivity against many viruses circulating in the 2024/2025 season [24]. Despite the antigenic difference between A H3N2 viruses in circulation and the vaccine strains, and in concordance with results from the EU and Canada, we did not observe relevant differences in aIVE between A H1N1pdm09 and A H3N2 in the primary care setting [8, 17]. We found, however, the lowest aIVE against A H3N2 in the hospital setting, though we lacked precision in the estimate due to the low number of events.

### 4.3 Strengths and Limitations

To estimate IVE in the primary care setting we used a well‐established sentinel system for virological surveillance, consisting of general practitioners and pediatricians in an important number of Italian regions [12]. Despite the large number of clinicians across the country contributing to the primary care surveillance network, our IVE estimates, especially in the stratified analysis, lacked precision due to the low number of tests performed. The lack of precision was more relevant for the hospital setting analysis, despite a larger number of tests carried out, due to limited data completeness in key variables preventing us from including most of the records. Limitation of power also hindered us from estimating IVE by vaccine type, which would have been important to inform public health policy. Another limitation is that we could not collect and link the single viral sequence information/molecular characteristics to each participant enrolled in the study.

One advantage of the test‐negative study design is that it helps control for confounding factors related to healthcare‐seeking behaviour, a common issue in observational studies, especially of generally non‐severe illnesses. The fundamental assumption is that the influenza vaccine influences the likelihood of influenza infections and/or hospitalisations, but not the risk of acquiring other ILIs [25]. Although we included in the multivariable analysis the main confounders (i.e., age group, sex, presence of chronic conditions, previous vaccination against influenza and calendar time), there may be a residual bias due to unmeasured confounders such as those related to socio‐economic condition, social interaction and household characteristics.

## 5 Conclusion

In Italy, during the 2024/2025 influenza season, the recommended vaccines were effective in preventing influenza illness requiring primary care and hospital attendance. Effectiveness was higher against influenza B, which affected predominantly younger individuals, than against influenza A. We did not find relevant differences in vaccine effectiveness between influenza A H1N1pdm09 and A H3N2, but results should be interpreted with caution due to the lack of estimate precision.

## Author Contributions

**Daniel Cifo Arcos:** conceptualization, methodology, formal analysis, visualization, writing – original draft, writing – review and editing. **Alberto Mateo‐Urdiales:** conceptualization, methodology, formal analysis, visualization, writing – original draft, writing – review and editing. **Antonino Bella:** conceptualization, methodology, data curation, writing – review and editing. **Simona Puzelli:** methodology, data curation, writing – review and editing. **Marzia Facchini:** methodology, data curation, writing – review and editing. **Sara Piacentini:** methodology, data curation, writing – review and editing. **Emanuela Giombini:** methodology, data curation, writing – review and editing. **Benedetta Bellini:** methodology, data curation, writing – review and editing. **Massimo Fabiani:** methodology, data curation, writing – review and editing. **Patrizio Pezzotti:** conceptualization, writing – review and editing. **Paola Stefanelli:** conceptualization, writing – review and editing. **Anna Teresa Palamara:** conceptualization, writing – review and editing. **Members of the RespiVirNet Regional Network:** data curation, writing – review and editing.

## Funding

The surveillance activities have been conducted with the support of the Italian Ministry of Health and partly by European Union funding within the Next Generation EU‐MUR PNRR Extended Partnership initiative on Emerging Infectious Diseases (Project no. PE00000007, INF‐ACT).

## Ethics Statement

No specific ethical approval was required for this study. The samples analysed were collected within routine respiratory virus surveillance activities.

## Conflicts of Interest

The authors declare no conflicts of interest.

## RespiVirNet Regional Network

### Laboratorio

Patrizia Falcone^4^, Lucia Collini^5^, Paolo Fazii^6^, Maria Chironna^7^, Francesca Greco^8^, Fabio Tramuto^9^, Valeria Ghisetti^10^, Andrea Orsi^11,12^, Elena Pariani^13^, Alberto Rizzo^14^, Fausto Baldanti^15,16^, Elisabetta Pagani^17^, Fabio Barbone^18^, Valerio Corsini^19^, Barbara Camilloni^20^, Stefano Menzo^21^, Massimiliano Scutellà^22^, Teresa Lopizzo^23^, Maria Eugenia Colucci^24^, Simone Giannecchini^25^, Rosaria Santangelo^26^, Claudia Tiberio^27^, Claudia Del Vecchio^28^, Claudia Piu^29^

### Referenti regionali della sorveglianza

Hélène Imperial^30^, Giovanna Parrino^31^, Giulio Matteo^32^, Filippo Da Re^33^, Annalisa Finesso^34^, Barbara Rita Porchia^35^, Danilo Cereda^36^, Maria Grazia Zuccali^37^, Enrico Volpe^38^, Fabio Filippetti^39^, Federico Grammatico^40^, Pierina Tanchis^41^, Manuela Mariano^42^, Giovanni Canitano^43^, Vincenzo Giordano^44^, Cristina Zappetti^45^, Simona Foresi^46^, Silvia Spertini^47^, Francesco Lucia^48^, Adriano Murgano^49^, Cinzia Annatea Germinario^50^

^1^S.C. Medicina di LaboratorioOspedale Regionale Umberto PariniAosta, Italy

^2^Ospedale S. Chiara, Azienda Provinciale per i Servizi Sanitari, Provincia Autonoma di TrentoItaly

^3^UOC di “Microbiologia e Virologia Clinica a valenza regionale”PO “Spirito Santo”Pescara, Italy

^4^Laboratorio di Epidemiologia molecolare e Sanità Pubblica‐UOC IgieneAOUC Policlinico di BariItaly

^5^UOC di “Microbiologia e Virologia”, AO AnnunziataCosenza, Italy

^6^UOC Epidemiologia Clinica con Registro Tumori ‐AOU Policlinico “Paolo Giaccone”, Palermo, Italy

^7^Laboratorio di Virologia e MicrobiologiaOspedale Amedeo di Savoia‐ASL Città di TorinoTurin, Italy

^8^Department of Health Sciences (DISSAL), University of GenoaGenoa, Italy

^9^Hygiene UnitIRCCS San Martino Polyclinic HospitalGenoa, Italy

^10^Department of biomedical sciences for healthUniversity of MilanMilan, Italy

^11^Clinical Microbiology, Virology and BioemergenciesL. Sacco University Hospital, ASST Fatebenefratelli‐SaccoMilan, Italy

^12^Department of Clinical, Surgical, Diagnostic and Paediatric SciencesUniversity of PaviaPavia, Italy

^13^SC Microbiology and VirologyIRCCS Policlinico San MatteoPavia, Italy

^14^Laboratory of Microbiology and VirologyProvincial Hospital of Bolzano (SABES‐ASDAA), Lehrkrankenhaus der Paracelsus Medizinischen Privatuniversität, Bolzano, Italy

^15^Department of Medicine, Surgery and Health SciencesUniversity of TriesteTrieste, Italy

^16^Virology DivisionPisa University HospitalPisa, Italy

^17^Microbiology and Clinical Microbiology, Department of Medicine and SurgeryUniversity of PerugiaPerugia, Italy

^18^Dipartimento di Scienze Biomediche e Sanità PubblicaUniversità Politecnica delle MarcheAncona, Italy

^19^UOSVD Microbiologia e Diagnostica Molecolare Avanzata P.O. A. Cardarelli Campobasso Azienda Sanitaria Regionale MoliseCampobasso, Italy

^20^UOS Microbiologia e VirologiaAOR San CarloPotenza, Italy

^21^Laboratorio di Igiene e Sanità Pubblica, Dipartimento di Medicina e ChirurgiaUniversità di ParmaParma, Italy

^22^Department of Experimental and Clinical Medicine University of Florence, SOD Microbiology and VirologyAOUCFlorence, Italy

^23^Dipartimento di Scienze di Laboratorio ed EmatologicheFondazione Policlinico Universitario A. Gemelli IRCCSRome, Italy

^24^U.O.C Microbiologia e VirologiaA.O.R.N Dei colli NapoliNaples, Italy

^25^Laboratorio di Virologia, Dipartimento di Medicina MolecolareUniversità degli Studi di Padova

^26^Laboratorio di Virologia della S.C. di Microbiologia e Virologia dell'AOU di SassariItaly

^27^Azienda USLAosta, Italy

^28^Dipartimento per le Attività Sanitarie e Osservatorio Epidemiologico, Assessorato della Salute, Regione SicilianaPalermo, Italy

^29^Settore Prevenzione collettiva e Sanità pubblica, Direzione Generale Cura della persona, salute e welfare, Emilia‐Romagna RegionBologna, Italy

^30^Direzione Prevenzione Sicurezza alimentare, VeterinariaVeneto

^31^Piedmont Regional Service for the Epidemiology of Infectious Diseases (SeREMI)‐Research and Innovation Department (DAIRI), “SS Antonio e Biagio e C. Arrigo” University Hospital, Alessandria, Italy

^32^Settore Prevenzione, salute e sicurezza, veterinaria‐Direzione Sanità, Welfare e Coesione Sociale, Regione ToscanaFlorence, Italy

^33^Directorate General for Health, Lombardy RegionMilan, Italy

^34^Dipartimento di Prevenzione, Centro per i servizi sanitariTrentoItaly

^35^Direzione Regionale Salute e Integrazione Sociosanitaria, Regione LazioRoma, Italy

^36^Agenzia Regionale Sanitaria, Regione MarcheItaly

^37^S.C. Coordinamento Regionale delle attività di Prevenzione e di Epidemiologia, Dipartimento Prevenzione, Epidemiologia, Programmazione e Controlli, Regione LiguriaItaly

^38^Direzione Generale della Sanità, Regione SardegnaCagliari, Italy

^39^Azienda Sanitaria Regionale del MoliseCampobasso, Italy

^40^Direzione Generale per la Salute e le Politiche della Persona, Regione BasilicataItaly

^41^UOD 02 Prevenzione e Sanità Pubblica, Regione CampaniaNaples, Italy

^42^Direzione centrale salute, politiche sociali e disabilità, Regione Friuli Venezia GiuliaUdine, Italy

^43^Regione UmbriaPerugia, Italy

^44^Servizio Igiene e Sanità Pubblica, Azienda Sanitaria Alto AdigeBolzano, Italy

^45^Dipartimento Tutela della Salute regione Calabria, Regione CalabriaCatanzaro, Italy

^46^Servizio Prevenzione Sanitaria, Medicina Territoriale del Dipartimento Sanità Regione AbruzzoPescara, Italy

^47^Dipartimento Interdisciplinare di MedicinaUniversity of BariBari, Italy

## Supporting information


**Table S1:** Number and percentage of clinicians taking part in the sentinel virological surveillance, and tests carried out during the season, by region in Italy, 2024–2025
**Table S2:** Number and percentage of tests carried out in hospital setting during the season, by region in Italy, 2024–2025
**Table S3:** Crude and adjusted vaccine effectiveness against any influenza virus, and by virus type and subtype. Overall and stratified by age group in Italy, during the 2024/2025 season. Excluding previous vaccination status

## Data Availability

The data that support the findings of this study are available on request from the corresponding author. The data are not publicly available due to privacy or ethical restrictions.
