# Correction to “Effectiveness of Influenza Vaccines in Italy in the 2024/2025 Season: A Nationwide, Test‐Negative Design Study Based on Surveillance Records”

**DOI:** 10.1111/irv.70275

**Published:** 2026-06-03

Cifo, D. , Mateo‐Urdiales, A. , Puzelli, S. , Facchini, M. , Piacentini, S. , Giombini, E. , Bellini, B. , Fabiani, M. , Pezzotti, P. , Stefanelli, P. , Palamara, A. , Bella, A. and the RespiVirNet network (2026), Effectiveness of Influenza Vaccines in Italy in the 2024/2025 Season: A Nationwide, Test‐Negative Design Study Based on Surveillance Records. Influenza Other Respi Viruses, 20: e70252, 10.1111/irv.70252.PMC1313001942059245

In “RespiVirNet Regional Network,” the affiliation numbers have been updated. It should read as follows:

4 S.C. Medicina di Laboratorio, Ospedale Regionale Umberto Parini, Aosta, Italy
5 Ospedale S. Chiara, Azienda Provinciale per i Servizi Sanitari, Provincia Autonoma di Trento, Italy
6 UOC di “Microbiologia e Virologia Clinica a valenza regionale”, PO “Spirito Santo”, Pescara, Italy
7 Laboratorio di Epidemiologia molecolare e Sanità Pubblica ‐ UOC Igiene, AOUC Policlinico di Bari, Italy
8 UOC di" Microbiologia e Virologia", AO Annunziata, Cosenza, Italy
9 UOC Epidemiologia Clinica con Registro Tumori ‐ AOU Policlinico "Paolo Giaccone", Palermo, Italy
10 Laboratorio di Virologia e Microbiologia, Ospedale Amedeo di Savoia ‐ ASL Città di Torino, Turin, Italy
11 Department of Health Sciences (DISSAL), University of Genoa, Genoa, Italy
12 Hygiene Unit, IRCCS San Martino Polyclinic Hospital, Genoa, Italy
13 Department of biomedical sciences for health, University of Milan, Milan, Italy
14 Clinical Microbiology, Virology and Bioemergencies, L. Sacco University Hospital, ASST Fatebenefratelli‐Sacco, Milan, Italy
15 Department of Clinical, Surgical, Diagnostic and Paediatric Sciences, University of Pavia, Pavia, Italy
16 SC Microbiology and Virology, IRCCS Policlinico San Matteo, Pavia, Italy
17 Laboratory of Microbiology and Virology, Provincial Hospital of Bolzano (SABES‐ASDAA), Lehrkrankenhaus der Paracelsus Medizinischen Privatuniversität, Bolzano, Italy
18 Department of Medicine, Surgery and Health Sciences, University of Trieste, Trieste, Italy
19 Virology Division, Pisa University Hospital, Pisa, Italy
20 Microbiology and Clinical Microbiology, Department of Medicine and Surgery, University of Perugia, Perugia, Italy
21 Dipartimento di Scienze Biomediche e Sanità Pubblica, Università Politecnica delle Marche, Ancona, Italy
22 UOSVD Microbiologia e Diagnostica Molecolare Avanzata P.O. A. Cardarelli Campobasso Azienda Sanitaria Regionale Molise, Campobasso, Italy
23 UOS Microbiologia e Virologia, AOR San Carlo, Potenza, Italy
24 Laboratorio di Igiene e Sanità Pubblica, Dipartimento di Medicina e Chirurgia, Università di Parma, Parma, Italy
25 Department of Experimental and Clinical Medicine University of Florence, SOD Microbiology and Virology, AOUC, Florence, Italy
26 Dipartimento di Scienze di Laboratorio ed Ematologiche, Fondazione Policlinico Universitario A. Gemelli IRCCS, Rome, Italy
27 U.O.C Microbiologia e Virologia A.O.R.N Dei colli Napoli, Naples, Italy
28 Laboratorio di Virologia, Dipartimento di Medicina Molecolare, Università degli Studi di Padova
29 Laboratorio di Virologia della S.C. di Microbiologia e Virologia dell'AOU di Sassari, Italy
30 Azienda USL, Aosta, Italy
31 Dipartimento per le Attività Sanitarie e Osservatorio Epidemiologico, Assessorato della Salute, Regione Siciliana, Palermo, Italy
32 Settore Prevenzione collettiva e Sanità pubblica, Direzione Generale Cura della persona, salute e welfare, Emilia‐Romagna Region, Bologna, Italy
33 Direzione Prevenzione Sicurezza alimentare, Veterinaria, Veneto
34 Piedmont Regional Service for the Epidemiology of Infectious Diseases (SeREMI) ‐ Research and Innovation Department (DAIRI), "SS Antonio e Biagio e C. Arrigo" University Hospital, Alessandria, Italy
35 Settore Prevenzione, salute e sicurezza, veterinaria ‐ Direzione Sanità, Welfare e Coesione Sociale, Regione Toscana, Florence, Italy
36 Directorate General for Health, Lombardy Region, Milan, Italy
37 Dipartimento di Prevenzione, Centro per i servizi sanitari, Trento, Italy
38 Direzione Regionale Salute e Integrazione Sociosanitaria, Regione Lazio, Roma, Italy
39 Agenzia Regionale Sanitaria, Regione Marche, Italy
40 S.C. Coordinamento Regionale delle attività di Prevenzione e di Epidemiologia, Dipartimento Prevenzione, Epidemiologia, Programmazione e Controlli, Regione Liguria, Italy
41 Direzione Generale della Sanità, Regione Sardegna, Cagliari, Italy
42 Azienda Sanitaria Regionale del Molise, Campobasso, Italy
43 Direzione Generale per la Salute e le Politiche della Persona, Regione Basilicata, Italy
44 UOD 02 Prevenzione e Sanità Pubblica, Regione Campania, Naples, Italy
45 Direzione centrale salute, politiche sociali e disabilità, Regione Friuli Venezia Giulia, Udine, Italy
46 Regione Umbria, Perugia, Italy
47 Servizio Igiene e Sanità Pubblica, Azienda Sanitaria Alto Adige, Bolzano, Italy
48 Dipartimento Tutela della Salute regione Calabria, Regione Calabria, Catanzaro, Italy
49 Servizio Prevenzione Sanitaria, Medicina Territoriale del Dipartimento Sanità Regione Abruzzo, Pescara, Italy
50 Dipartimento Interdisciplinare di Medicina, University of Bari, Bari, Italy

We apologize for this error.

