# Supplementary material for: Effectiveness of Influenza Vaccines in Italy in the 2024/2025 Season: A Nationwide, Test‐Negative Design Study Based on Surveillance Records
Source: Influenza Other Respir Viruses. 2026 Apr 30;20(5):e70252. doi: 10.1111/irv.70252 (PMC13130019; doi:10.1111/irv.70252)
Supplement: Supplementary file 1 — Table S1: Number and percentage of clinicians taking part in the sentinel virological surveillance, and tests carried out during the season, by region in Italy, 2024–2025 Table S2: Number and percentage of tests carried out in hospital setting during the season, by region in Italy, 2024–2025 Table S3: Crude and adjusted vaccine effectiveness against any influenza virus, and by virus type and subtype. Overall and stratified by age group in Italy, during the 2024/2025 season. Excluding previous vaccination status [file IRV-20-e70252-s001.docx]

Supplementary material.

**Table S1. Number and percentage of clinicians taking part in the sentinel virological surveillance, and tests carried out during the season, by region in Italy, 2024-2025**

| **Region** | **Clinicians**  **N (%)** | **Tests**  **N (%)** |
| --- | --- | --- |
| Abruzzo | 30 (7.4%) | 446 (4.1%) |
| Emilia-Romagna | 91 (22.5%) | 2115 (19.5%) |
| Friuli-Venezia Giulia | 28 (6.9%) | 1075 (9.9%) |
| Lombardia | 98 (24.3%) | 3263 (30.1%) |
| Provincia Autonoma di Bolzano/Bozen | 1 (0.2%) | 50 (0.5%) |
| Puglia | 83 (20.5%) | 407 (3.8%) |
| Sicilia | 26 (6.4%) | 1454 (13.4%) |
| Umbria | 3 (0.7%) | 212 (2%) |
| Valle d'Aosta/Vallée d'Aoste | 11 (2.7%) | 385 (3.6%) |
| Veneto | 33 (8.2%) | 1417 (13.1%) |

**Table S2. Number and percentage of tests carried out in hospital setting during the season, by region in Italy, 2024-2025**

| **Region** | **Tests**  **N (%)** |
| --- | --- |
| Basilicata | 524 (15.9%) |
| Campania | 4 (0.1%) |
| Emilia-Romagna | 629 (19.1%) |
| Friuli-Venezia Giulia | 27 (0.8%) |
| Lazio | 2 (0.1%) |
| Lombardia | 32 (1%) |
| Marche | 208 (6.3%) |
| Molise | 5 (0.2%) |
| Provincia Autonoma di Bolzano/Bozen | 5 (0.2%) |
| Puglia | 1032 (31.4%) |
| Sardegna | 68 (2.1%) |
| Sicilia | 274 (8.3%) |
| Toscana | 310 (9.4%) |
| Valle d'Aosta/Vallée d'Aoste | 9 (0.3%) |
| Veneto | 159 (4.8%) |

**Table S3. Crude and adjusted vaccine effectiveness against any influenza virus, and by virus type and subtype. Overall and stratified by age group in Italy, during the 2024/25 season. Excluding previous vaccination status**

| **Primary Care** | **Age group (years)** | **IVE (crude)** | **95% CI** | **IVE (adj.)*** | **95% CI** |
| --- | --- | --- | --- | --- | --- |
| Any influenza virus | Total | 64.7 | 56.9 to 71.2 | 68.1 | 60.6 to 74.3 |
|  | <18 | 67.5 | 57.4 to 75.5 | 72.9 | 64.3 to 79.8 |
|  | 18-64 | 54.3 | 34.6 to 68.9 | 60.1 | 41.9 to 72.6 |
|  | 65+ | 56.4 | 25.7 to 75.3 | 59.9 | 28.9 to 77.4 |
| Influenza A H1N1pdm09 | Total | 45.5 | 27.5 to 59.9 | 48.2 | 29.7 to 62.6 |
|  | <18 | 37.6 | 9.3 to 58.6 | 43.9 | 17.2 to 63.3 |
|  | 18-64 | 38.0 | -3 to 65.4 | 44.8 | 4.3 to 68.2 |
|  | 65+ | 71.6 | 34.5 to 89.6 | 69.8 | 23.3 to 88.1 |
| Influenza A H3N2 | Total | 47.7 | 28.4 to 62.8 | 55.6 | 38.2 to 68.8 |
|  | <18 | 50.1 | 22.4 to 69.7 | 59.0 | 35.8 to 75.2 |
|  | 18-64 | 56.3 | 18.5 to 79.5 | 63.4 | 27.1 to 81.6 |
|  | 65+ | 19.0 | -67.9 to 62.5 | 24.9 | -64.3 to 65.6 |
| Influenza B | Total | 84.8 | 77.7 to 90.1 | 85.9 | 79.2 to 90.9 |
|  | <18 | 89.0 | 81.2 to 94.2 | 91.1 | 84.7 to 95.3 |
|  | 18-64 | 65.8 | 39.4 to 82.7 | 69.5 | 43 to 83.7 |
|  | 65+ | 68.5 | -24.7 to 95.2 | 75.4 | -23.5 to 95.1 |

| **Hospital setting** | **Age group (years)** | **IVE (crude)** | **95% CI** | **IVE (adj.)*** | **95% CI** |
| --- | --- | --- | --- | --- | --- |
| Any influenza virus | Total | 51.3 | 33 to 65.4 | 64.7 | 50 to 75.6 |
|  | <18 | 77.0 | 56.3 to 89.3 | 80.8 | 63 to 91.2 |
|  | 18-64 | 71.3 | 18.3 to 93.2 | 74.5 | 14.8 to 92.4 |
|  | 65+ | 40.4 | 9 to 61.7 | 48.6 | 18.9 to 67.4 |
| Influenza A H1N1pdm09 | Total | 41.8 | 5.5 to 66.4 | 63.2 | 37.6 to 79.5 |
|  | <18 | 49.2 | -26.3 to 84.8 | 49.2 | -30.8 to 85.1 |
|  | 18-64 | - | - | 100.0 | -Inf to 100 |
|  | 65+ | 56.5 | 21.3 to 77.6 | 60.1 | 23.7 to 79.1 |
| Influenza A H3N2 | Total | 29.9 | -4.8 to 54.9 | 51.2 | 24.4 to 69.5 |
|  | <18 | 73.2 | 34.6 to 91.9 | 77.8 | 44.7 to 93.4 |
|  | 18-64 | 37.2 | -81.6 to 85.2 | 45.2 | -86.9 to 83.9 |
|  | 65+ | 16.1 | -42.5 to 52.4 | 32.8 | -18.4 to 61.9 |
| Influenza B | Total | 95.8 | 81.3 to 99.8 | 95.7 | 80.2 to 99.8 |
|  | <18 | 100.0 | 90 to 100 | 100.0 | 100 to 100 |
|  | 18-64 | - | - | - | - to - |
|  | 65+ | 50.3 | -195.6 to 97.4 | 42.8 | -396 to 93.4 |

*Adjusted for age, sex, calendar time and presence of comorbidities
